# Rare central venous catheter malposition – an ultrasound-guided approach would be helpful: a case report

**DOI:** 10.1186/s13256-016-1026-0

**Published:** 2016-09-13

**Authors:** Keisuke Kumada, Nobuo Murakami, Hideshi Okada, Izumi Toyoda, Shinji Ogura, Hiroshi Kondo, Atsuhiro Fukuda

**Affiliations:** 1 Patient Safety Division, Gifu University Hospital, Yanaido1-1, Gifu, ZC: 501-1194 Japan; 2 Department of Emergency & Disaster Medicine, Gifu University School of Medicine, Gifu, Japan; 3 Department of Radiology, Teikyo University Hospital, Tokyo, Japan; 4 Shonan Kamakura General Hospital, Kanagawa, Japan

**Keywords:** Central venous catheter, Malposition, Internal mammary artery, Adverse event

## Abstract

**Background:**

A central venous catheter enables the measurement of hemodynamic variations, such as accurate central venous pressure; catheter malposition may induce potentially fatal complications. This case report describes a rare central venous catheter tip malposition in the right internal mammary artery.

**Case presentation:**

A 56-year-old Japanese woman who presented with severe pneumonia secondary to scleroderma was treated under ventilator support because of acute respiratory failure. A right central venous catheter was inserted using a landmark technique to monitor central venous pressure and administer medications. However, central venous waveforms detected by the catheter using a pressure lot transducer were later found to be absent. Further imaging studies, including plain radiography, computed tomography, and angiography, confirmed central venous catheter malposition in the internal mammary artery. Her right internal mammary artery was embolized using two interlocking detachable coils, and the central venous catheter was removed from her internal mammary artery without further complications.

**Conclusions:**

Internal mammary artery malposition is a rare but potentially lethal complication of central venous catheter catheterization; however, caution should be taken regarding the assessment of risk factors and management of a severe complication. An ultrasound-guided approach would be helpful.

## Background

A central venous catheter (CVC) enables the measurement of hemodynamic variations, including accurate measurement of central venous pressure (CVP), as well as delivery of medications and nutritional support. Numerous complications of central venous catheterization have been reported, including malposition, arterial puncture, hematoma, pneumothorax, hemothorax, infection, and thrombosis. Catheter malposition may result in severe complications [1]. We report a rare case of a CVC tip malpositioned in the right internal mammary artery (IMA).

## Case presentation

A 56-year-old Japanese woman who presented with severe pneumonia secondary to scleroderma was treated under ventilator support because of acute respiratory failure. A physical examination revealed fever with a body temperature of 100.7 °F (38.2 °C), tachypnea with a respiratory rate of 25 breaths/minute, blood pressure of 127/67 mmHg, pulse 120 beats/minute, and level of consciousness was normal. Arterial blood gas analysis revealed pH 7.419, partial pressure of carbon dioxide in arterial blood (PaCO_2_) 55.9 mmHg, partial pressure of oxygen in arterial blood (PaO_2_) 64.5 mmHg, and bicarbonate (HCO_3_^-^) 40.9 mmol/L. A laboratory test revealed the following data: leukocytosis at 20.6×10^−3^/μL, hemoglobin level of 8.5 g/dL, platelet count of 331×10^−3^/μL, an international normalized ratio (INR) of 1.64, serum creatinine level of 0.18 mg/dL, C-reactive protein level of 23.44 mg/dL, serum gamma-glutamyltransferase level of 139 IU/L, alkaline phosphatase level of 618 IU/L, and total bilirubin of 0.4 mg/dL. She had no prior relevant medical or surgical history.

**Fig. 1:**
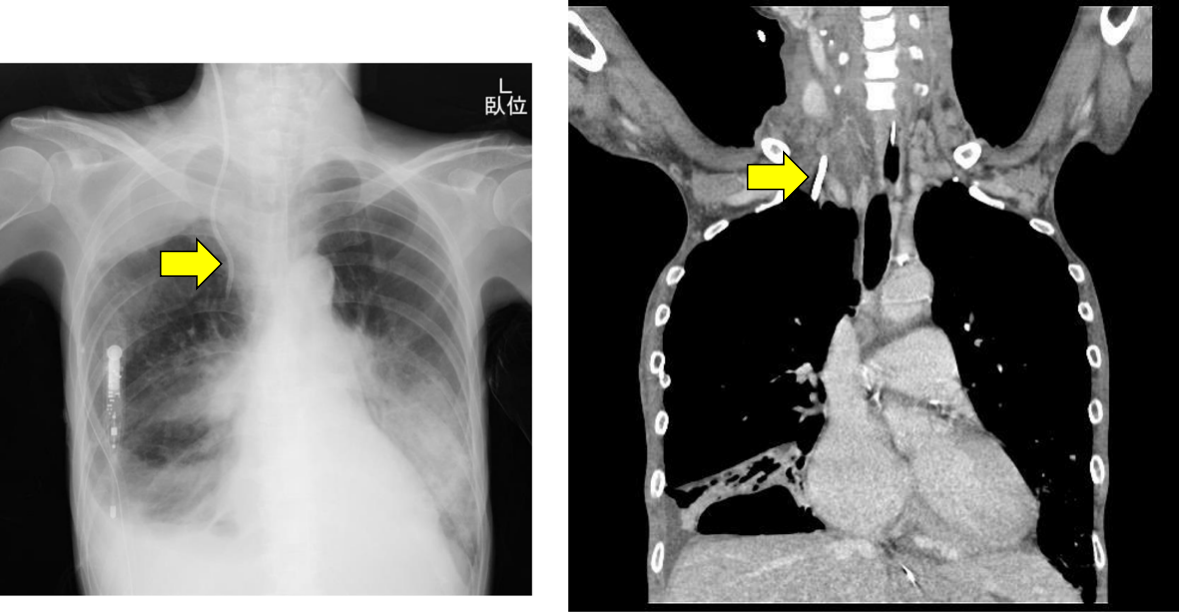
Chest X–ray revealing a CVC descending lateral to the mediastinal margin (*left arrow*). Thoracic computed tomography demonstrated CVC malpositioning in the right IMA (*right arrow*)

**Fig. 2:**
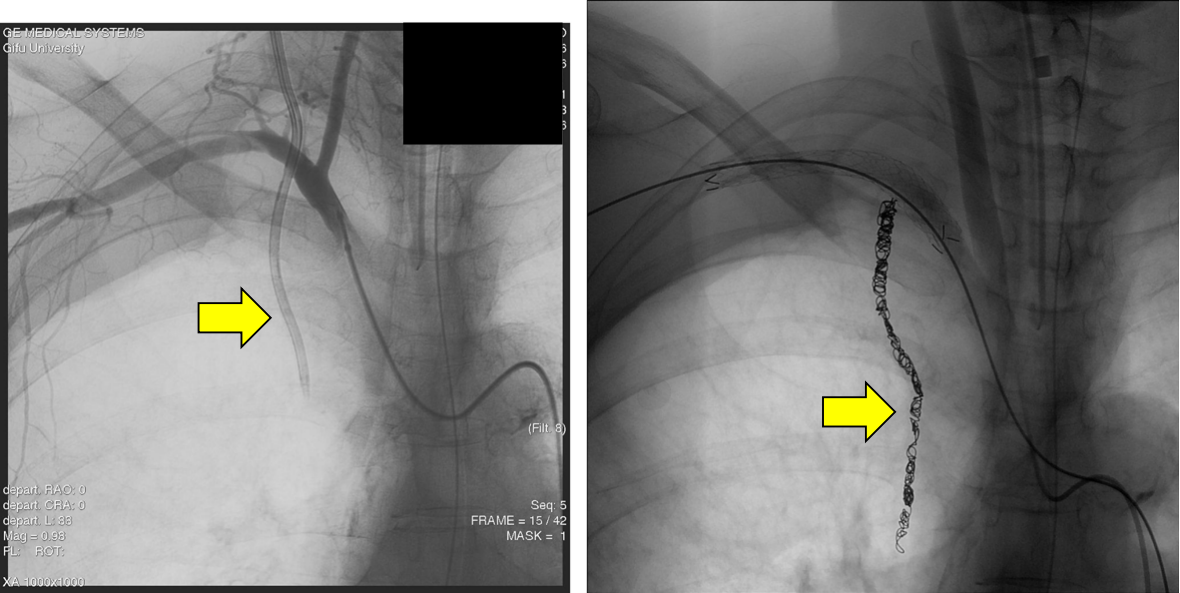
Right branchiocephalic arteriography revealing catheter placement in the right internal mammary artery (*left arrow*). Right internal mammary artery occlusion following coil embolization (*right arrow*)

A right CVC was inserted using a landmark technique to monitor CVP and administer medications. A double lumen 16 G catheter was inserted by an experienced internal physician using the Seldinger technique. With this approach, a resistance to guidewire advancement was met at a depth of 7 cm, but it was advanced further without resistance. CVC was fixed at 13 cm at the skin level, after free aspiration of blood. Continuous infusion of normal saline through CVC was initiated using an infusion pump. CVP and arterial waveforms were not observed following connection of the catheter to a pressure kit transducer. A chest X-ray was immediately arranged to confirm the catheter position (Fig. 1). The catheter was found to have descended lateral to the right mediastinal margin without signs of either pneumothorax or pleural effusion. Thoracic computed tomography findings were suggestive of CVC malposition in her right IMA (Fig. 1). An angiography performed using CVC confirmed the malposition in her right IMA (Fig. 2 left). Selective right subclavian and right internal angiographies were performed with consideration of the injury to her right subclavian artery. Subsequently, embolization of her right IMA was performed with two interlocking detachable coils (IDC; Boston Scientific Japan, Tokyo, Japan). After coil embolization, her right IMA was occluded before successful removal of the catheter without further complications, such as acute bleeding (Fig. 2 right). A few days later, she developed acute respiratory distress syndrome, and she died on the seventh day of intensive care unit (ICU) admission.

## Discussion

**Fig. 3:**
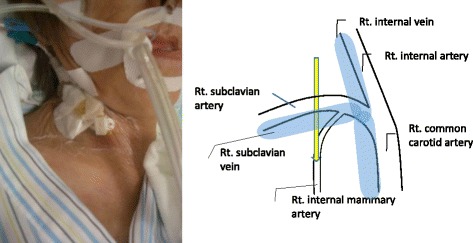
Schematic depiction of the anatomy of the right internal mammary artery in relation to the right subclavian artery and the internal artery (*right*). The mechanism of the malposition is not entirely clear. It is possible that the catheter was inserted too proximally to the optimal insertion site (*left*) so that it was directly inserted into the internal mammary artery (*right*, *arrow*). *Rt.* right

Catheter malpositioning may result in potentially fatal complications. The incidence of catheter malposition has been reported to be between 5 and 12 % [2]. In a large prospective study by Pikwer *et al*. of 1619 central line catheterizations [3], using the landmark technique, cannulation by the right subclavian vein was associated with the highest risk of malposition, 9.1 %, compared with 1.4 % by the right internal jugular vein. There have been a number of previous reports of CVC malpositioning in the internal mammary vein [4–8]. However, IMA malpositioning is extremely rare with only a few reports of this complication, all involving an inadvertent puncture of IMA [9–11]. Puncture of IMA may induce mediastinal or pleural hematoma, pseudoaneurysm, or arteriovenous fistula. Arterial bleeding can take a dramatic course with severe bleeding inducing hemomediastinum or hemothorax, thus requiring immediate treatment. In this case, no signs of inadvertent arterial puncture of IMA were observed; however, the possibility for massive bleeding occurred when the malpositioned catheter was removed. The mechanism of IMA malpositioning is unclear. The bevel orientation facilitates the progression of the guidewire in the intended direction; incorrect venipuncture positioning may have caused catheter insertion inferior to the recommended site (Fig. 3 left). The catheter was directly inserted into IMA without injury to the subclavian artery (Fig. 3 right). Catheter malpositioning was adequately demonstrated by selective angiography, with subsequent embolization leading to a satisfactory result. The micro-catheter system allowed selective catheterization and embolization of IMA. Transarterial embolization with microcoils is an efficient technique for the treatment of catheter malpositioning.

This case highlights the importance of CVP waveform monitoring and chest X-ray in ensuring correct catheter placement, particularly in unconscious or intubated patients in intensive care settings. CVP waveform could not be obtained, which strongly indicated the possibility of misplacement of CVC despite free aspiration of blood. Therefore, pressure waveform monitoring and checking and acting on chest X-ray after central catheter placement may be used to prevent accidental arterial cannulation. Catheter malpositioning occurred in this case despite cannulation being performed by an experienced internal physician. The experience of the physician inserting a CVC is of paramount importance. The risk of mechanical complications increases significantly if the doctor performing the procedure has inserted less than 50 CVCs [12]. Simulation training and use of ultrasound guidance were associated with improved in-hospital performance of CVC insertion [13]. Ultrasound guidance, in addition to direct visualization, when placing venous catheters has been shown to reduce the incidence of complications and is useful in identifying diminutive or thrombosed vessels [14]. Ultrasound guidance has previously been recommended for all CVC procedures [15]. IMA malposition is a rare but potentially lethal complication of CVC that should be considered in risk factor assessments and the management of severe complications resulting from CVC.

## Conclusions

We present a rare case of CVC malpositioning in the right IMA. Awareness of the possibility of this rare complication and early angiographic intervention may avoid further complications. Therefore, one must consider the possibility of incorrect placement of the catheter. Monitoring the central venous wave form and obtaining chest X-rays can be useful methods to confirm the position of the catheter. An ultrasound-guided approach rather than the use of a landmark technique to insert CVC will help. Ultrasound guidance is important to minimize the risk of malposition.

## Abbreviations

CVC: Central venous catheter
CVP: Central venous pressure
IMA: Internal mammary artery
